# Cartilage Regeneration in Osteoarthritic Patients by a Composite of Allogeneic Umbilical Cord Blood‐Derived Mesenchymal Stem Cells and Hyaluronate Hydrogel: Results from a Clinical Trial for Safety and Proof‐of‐Concept with 7 Years of Extended Follow‐Up

**DOI:** 10.5966/sctm.2016-0157

**Published:** 2016-09-09

**Authors:** Yong‐Beom Park, Chul‐Won Ha, Choong‐Hee Lee, Young Cheol Yoon, Yong‐Geun Park

**Affiliations:** ^1^ Department of Orthopedic Surgery, Chung‐Ang University Hospital, Chung‐Ang University College of Medicine, Seoul, Republic of Korea; ^2^ Department of Orthopedic Surgery, Samsung Medical Center, Sungkyunkwan University School of Medicine, Seoul, Republic of Korea; ^3^ Stem Cell & Regenerative Medicine Research Institute, Samsung Medical Center, Sungkyunkwan University School of Medicine, Seoul, Republic of Korea; ^4^ Department of Health Sciences and Technology, Samsung Advanced Institute of Health Sciences and Technology, Sungkyunkwan University, Seoul, Republic of Korea; ^5^ Department of Radiology, Samsung Medical Center, Sungkyunkwan University School of Medicine, Seoul, Republic of Korea; ^6^ Department of Orthopedic Surgery, Jeju National University Hospital, Jeju National University School of Medicine, Jeju, Republic of Korea

**Keywords:** Osteoarthritis, Cartilage regeneration, Mesenchymal stem cells, Human umbilical cord blood, Hyaluronic acid

## Abstract

Few methods are available to regenerate articular cartilage defects in patients with osteoarthritis. We aimed to assess the safety and efficacy of articular cartilage regeneration by a novel medicinal product composed of allogeneic human umbilical cord blood‐derived mesenchymal stem cells (hUCB‐MSCs). Patients with Kellgren‐Lawrence grade 3 osteoarthritis and International Cartilage Repair Society (ICRS) grade 4 cartilage defects were enrolled in this clinical trial. The stem cell‐based medicinal product (a composite of culture‐expanded allogeneic hUCB‐MSCs and hyaluronic acid hydrogel [Cartistem]) was applied to the lesion site. Safety was assessed by the World Health Organization common toxicity criteria. The primary efficacy outcome was ICRS cartilage repair assessed by arthroscopy at 12 weeks. The secondary efficacy outcome was visual analog scale (VAS) score for pain on walking. During a 7‐year extended follow‐up, we evaluated safety, VAS score, International Knee Documentation Committee (IKDC) subjective score, magnetic resonance imaging (MRI) findings, and histological evaluations. Seven participants were enrolled. Maturing repair tissue was observed at the 12‐week arthroscopic evaluation. The VAS and IKDC scores were improved at 24 weeks. The improved clinical outcomes were stable over 7 years of follow‐up. The histological findings at 1 year showed hyaline‐like cartilage. MRI at 3 years showed persistence of the regenerated cartilage. Only five mild to moderate treatment‐emergent adverse events were observed. There were no cases of osteogenesis or tumorigenesis over 7 years. The application of this novel stem cell‐based medicinal product appears to be safe and effective for the regeneration of durable articular cartilage in osteoarthritic knees. Stem Cells Translational Medicine *2017;6:613–621*

**Significance Statement**

This paper reports the results of the first‐in‐human clinical trial investigating a stem cell‐based medicinal product called Cartistem (a composite of culture‐expanded allogeneic human umbilical cord blood‐derived mesenchymal stem cells and hyaluronic acid hydrogel). The application of the stem cell‐based medicinal product showed promising efficacy in terms of durable cartilage regeneration. Clinical outcomes were improved and stable, and no significant adverse events were observed over 7 years of follow‐up. There are currently no effective regenerative options for cartilage loss in osteoarthritis; thus, the researchers believe that this stem cell‐based medicinal product could provide a novel therapeutic option to regenerate worn cartilage lesions in osteoarthritis. The results of this early phase clinical trial warrant further investigation with a larger number of patients.

## Introduction

Articular cartilage has very limited capacity for self‐regeneration 1. Current treatment of cartilage defects in osteoarthritic patients is primarily palliative and includes medication and activity modification. The microfracture technique is applicable for repair of small‐ to mid‐sized cartilage defects in osteoarthritis, but results are suboptimal and cartilage tends to deteriorate within a few years 2, 3, 4. The application of autologous chondrocyte implantation (ACI) to osteoarthritic knees is not routinely recommended in elderly patients because of accelerated cellular senescence and dedifferentiation of autologous chondrocytes during the culture expansion process 5.

The use of stem cells has become a major interest in the field of regenerative medicine. Many human tissues, including bone marrow, adipose tissue, umbilical cord blood, and synovium, are well‐known sources of adult mesenchymal stem cells (MSCs) 6. However, the effects of MSCs on cartilage regeneration are still under investigation. Although some reports are indicated to have studied the application of stem cell therapy in osteoarthritis, they have predominantly been the result of medical procedures and are described as exploring the application of “a heterogeneous cell population obtained from an autologous tissue containing uncertain portions or number of stem cells” 7, 8, 9. There have been only a few reports on the application of autologous stem cells (a homogeneous stem cell population that was isolated from an autologous tissue and expanded in culture) for the regenerative treatment of osteoarthritic cartilage defects 10, 11, 12. The procedure of harvesting autologous tissue to obtain stem cells is invasive and requires the patient to undergo two operations. We could not find a report in the literature describing the application of allogeneic stem cells (a homogeneous stem cell population that was isolated form an allogeneic tissue and expanded in culture) for the repair of cartilage defects in osteoarthritic patients.

Human umbilical cord blood‐derived MSCs (hUCB‐MSCs) are isolated in a noninvasive manner and are also hypoimmunogenic 13. Furthermore, hUCB‐MSCs have shown high expansion capacity, which provides enough cells for therapeutic applications 14. Furthermore, we could not find any report in the literature on the long‐term safety and efficacy of allogeneic stem cells in human cartilage repair. Therefore, in this first‐in‐human clinical trial with extended duration of follow‐up, we aimed to investigate the safety and efficacy of a novel medicinal product (a composite of allogeneic hUCB‐MSCs and hyaluronic acid [HA] hydrogel [Cartistem; Medipost, Seongnam‐si, Gyeonggi‐do, Korea, http://www.medi‐post.com]) for articular cartilage regeneration in patients with osteoarthritic knees.

## Materials and Methods

### Study Design and Participants

This study was an open‐label, single‐arm, single‐center, phase I/II clinical trial with a 24‐week follow‐up period. In addition, we performed long‐term follow‐up to assess safety and efficacy over 7 years. Patients diagnosed with osteoarthritis of the knee joint with Kellgren‐Lawrence (K‐L) grade 3 and painful full‐thickness cartilage defects (International Cartilage Repair Society [ICRS] grade 4 lesions), which were not responsive to more than 6 months of palliative treatment, were eligible to participate. Other inclusion criteria were visual analog scale (VAS) score for pain between 40 and 60 mm during screening; a cartilage defect larger than 2 cm^2^; swelling, tenderness, and limited range of motion lower than grade 2; adequate blood coagulation activity; and adequate renal and hepatic function. Participants were excluded based on the following criteria: autoimmune or inflammatory joint disease; ligament instability higher than grade 2; a history of infection, surgery, or radiation therapy in the knee joint within the past 6 weeks; enrollment in any other clinical trial within the past 4 weeks; immunosuppressant use within the past 6 weeks; corticosteroid or viscosupplementation injection to the affected knee within the past 3 months; or current pregnancy or lactation.

Two groups were studied to ensure safety of the participants and to determine the maximum tolerated dose (MTD) based on dose‐limiting toxicity (DLT) of the study medicinal product (a composite of allogeneic hUCB‐MSCs and HA hydrogel). The first three participants were assigned to receive low‐dose MSCs (group A), and the next three were assigned to receive high‐dose MSCs (group B) (Table 1). One participant in the low‐dose group did not consent to 12‐week arthroscopy; thus one more participant was assigned to receive low‐dose MSCs (group A) to fulfill the requirement for proceeding to the high‐dose trial. The doses and cell concentrations were selected based on previous animal studies 15. The allogeneic hUCB‐MSCs were transplanted at a dose of 500 µl/cm^2^ of the defect area with a cell concentration of 0.5 × 10^7^ cells per milliliter. DLT was defined as any case with 2 or more of the following severe adverse reactions after transplantation: swelling, tenderness, limited range of motion, and pain of the knee joint. This study was reviewed and approved by the Korean Food and Drug Administration (FDA) and the institutional review board at our institution (Samsung Medical Center, Seoul, South Korea). Informed consent was obtained from all participants before enrollment in this study.

**Table 1:** Baseline and treatment characteristics of study participants

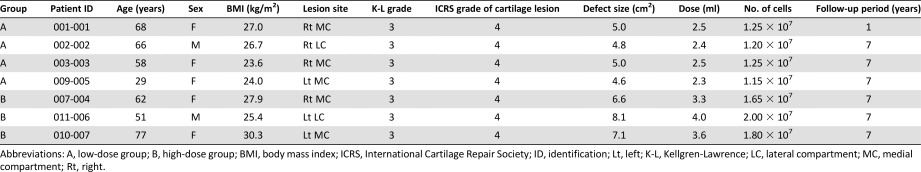

### Preparation of hUCB‐MSCs

The medicinal product (hUCB‐MSCs‐HA hydrogel composite) was produced under Good Manufacturing Practice guidelines, which were approved by the regulatory authority, and provided as an investigational new drug by Medipost. Human umbilical cord blood was collected from umbilical veins at the time of neonatal delivery, with informed consent from the mother, and stored in a cord blood bank. The hUCB‐MSCs were isolated and characterized according to previously published methods 16. Mononuclear cells were separated by density gradient centrifugation at 550*g* for 30 minutes using a Ficoll‐Hypaque solution (density, 1.077 g/ml; Sigma‐Aldrich, St. Louis, MO, http://www.sigmaaldrich.com). Isolated mononuclear cells were washed, suspended in minimum essential medium (a‐MEM; Thermo Fisher Scientific Life Sciences, Waltham, MA, http://www.thermofisher.com) supplemented with 10% fetal bovine serum (FBS; GE Healthcare Life Sciences, Pittsburgh, PA, http://www.gelifesciences.com), and then seeded into culture flasks at a concentration of 5 × 10^6^ cells per cm^2^. Cultures were maintained at 37°C in a humidified atmosphere containing 5% CO_2_, with a change of culture medium twice per week. Colonies of spindle‐shaped cells formed approximately 2 weeks after plating. Cells were trypsinized (0.25% trypsin; GE Healthcare Life Sciences), washed, and resuspended in culture medium (a‐MEM supplemented with 10% FBS) when the monolayer of MSC colonies reached 80% confluence.

### Application of the Stem Cell‐Based Medicinal Product

A standard arthroscopic examination was performed to assess cartilage defects. Then, the cartilage defect site was exposed through a small longitudinal arthrotomy. Multiple drill holes (5 mm in diameter and 5 mm deep) were made approximately 2–3 mm apart at the cartilage defect site of the femoral condyle. The study drug (Cartistem) was implanted in the drill holes of the lesion from the base to the surface (supplemental online Fig. 1). In the case of a kissing lesion, the composite was only transplanted in the femoral lesion because of dose limitation in terms of cell numbers. The wound was then closed and a splint was applied.

### Postoperative Rehabilitation

Patients were encouraged to perform quadriceps‐setting and straight leg‐raising exercises starting immediately after surgery. Active and active‐assisted range of motion exercise and nonweight‐bearing ambulation of the operated knee with a walking aid was started on postoperative day 1. Nonweight‐bearing ambulation was recommended for 12 weeks post‐transplantation to protect the repair tissue.

### Outcome Measures

Safety parameters included any abnormality found in the physical examination, vital signs, or laboratory studies as well as any adverse events (AEs). Adverse reactions in the knee joint were assessed by swelling, tenderness, active range of motion, and pain. Other AEs were categorized by the World Health Organization (WHO) Common Toxicity Criteria for Adverse Events 17. The primary efficacy parameter was the cartilage status at 12 weeks post‐transplantation, which was assessed by an arthroscopic examination according to the ICRS cartilage repair assessment 18. Secondary efficacy parameters were the 100‐mm VAS score for pain on walking and International Knee Documentation Committee (IKDC) subjective knee evaluation form score. Safety and efficacy parameters were assessed during screening, immediately after transplantation, and at 2, 4, 8, 12, and 24 weeks post‐transplantation per the initial protocol.

During the 7‐year extended follow‐up period, safety assessments, assessment of the VAS score for pain and IKDC score, arthroscopy, magnetic resonance imaging (MRI), and histological evaluations were performed. The safety parameters, VAS score for pain, and IKDC score were evaluated at 1‐, 3‐, and 7‐year follow‐up visits. An independent research assistant administered the questionnaires on safety and efficacy. Secondary arthroscopy was performed at 1 year, which was evaluated by an independent surgeon. Histological assessment was performed at 1 year. The biopsy specimens taken during the secondary arthroscopy were stained with Masson's trichrome, Safranin O, and immunohistochemical staining for type II collagen, and were evaluated by an independent pathologist blinded to this trial. A delayed gadolinium‐enhanced MRI of the cartilage (dGEMRIC) was performed at 3 years post‐transplantation to evaluate the quality of the regenerated cartilage. A dGEMRIC measures the T1 relaxation time of the cartilage and involves the intravenous administration of negatively charged contrast agent (Gd‐DTPA^2−^). The distribution of Gd‐DTPA^2‐^ is inversely proportional to glycosaminoglycan (GAG) content of the tissue of interest. Healthy cartilage, which contains an abundance of GAG, will show a low Gd‐DTPA^2−^ concentration, whereas GAG‐depleted degraded cartilage will show a high Gd‐DTPA^2−^ concentration. T1 relaxation times are inversely proportional to the concentration of Gd‐DTPA^2−^. The precontrast T1 relaxation time was calculated to evaluate the change in GAG content in the repair‐cartilage tissue. The T1 relaxation time was measured for 2 representative regions of interest (ROIs) in the same slice: the repair tissue area (ROI 1) and the healthy native cartilage (ROI 2). Quantitative R1, which represents the relaxation rate, is measured as 1/T1 (in 1/second). The change in R1 (ΔR1) was calculated as the difference between the precontrast and postcontrast R1. The ΔR1 represents the concentration of Gd‐DTPA^2−^. The ratio of ΔR1 in regenerated cartilage to ΔR1 in native cartilage is known as the relative ΔR1 index, which equals 1.0 in the case of perfect regeneration 19. The MRI scan was assessed by an independent musculoskeletal radiologist who was blinded to this study.

### Statistical Analysis

Safety analyses were performed on all of the participants who received hUCB‐MSCs‐HA hydrogel composite transplantation. Efficacy analyses included all participants who had at least one efficacy parameter evaluated. The pre‐ and posttransplantation VAS score for pain and IKDC subjective scores were compared using the Wilcoxon signed‐rank test. All statistical analyses were performed using SAS version 9.3 (SAS Institute, Cary, NC, http://www.sas.com). *P* values less than .05 were considered statistically significant.

## Results

### Baseline and Treatment Characteristics

Seven participants received a hUCB‐MSCs and HA hydrogel composite transplantation between November 2, 2005, and May 12, 2007 (supplemental online Fig. 2). The mean age of the participants was 58.7 years, and their mean BMI was 26.4 kg/m^2^. The average defect size was 4.9 cm^2^ in group A and 7.3 cm^2^ in group B. Group A participants were implanted with 1.15–1.25 × 10^7^ hUCB‐MSCs, and group B participants were implanted with 1.65–2.00 × 10^7^ hUCB‐MSCs according to the size of the articular cartilage defect. Six participants consented to join the extended follow‐up study after the 6‐month visit. One participant in group A opted out of the extended follow‐up study because she was satisfied with the current status of her knee and did not want additional examinations (Table 1).

### Safety and Toxicity

Mild to moderate AEs, but no serious AEs, were reported in five participants as follows: arthralgia, back pain, bladder distension, and elevated antithyroglobulin antibody level (Tables 2, 3). Only the elevated antithyroglobulin antibody level, which was classified as “increased infection susceptibility” according to the WHO criteria, was determined to be a treatment‐emergent adverse event (TEAE). An internal medicine specialist judged that it required no additional treatment, and it normalized spontaneously. No participant experienced DLT. Therefore, the higher dose was determined to be the MTD. In the extended follow‐up, no specific adverse reactions were observed in the six participants over 7 years. No participants underwent additional knee surgery or knee replacement conversion due to pain, aggravation, or functional impairment of the knee through the follow‐up period.

**Table 2:** Summary of treatment‐emergent adverse events by body system (all causalities)

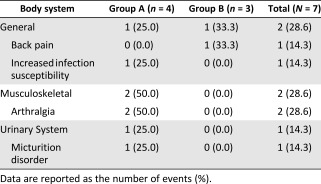

**Table 3:** Incidence of treatment‐emergent adverse events

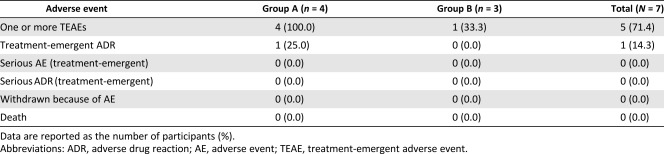

### Efficacy

**Figure 1:**
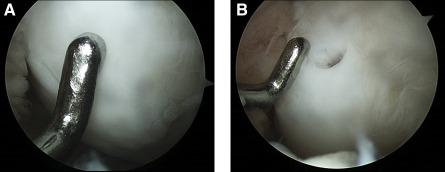
Findings from secondary arthroscopy at 1 year after human umbilical cord blood‐derived mesenchymal stem cell‐hyaluronic acid hydrogel composite transplantation. **(A):** Good resurfacing was identified at the defect site. The defect site was resurfaced well with regenerated cartilage showing a smooth surface. Firm consistency of the regenerated cartilage was palpable with a probe. Good integration with the surrounding cartilage was also observed. The repair cartilage was assessed as grade 2 according to the International Cartilage Repair Society assessment criteria in this participant (ID: 007‐004). **(B):** A biopsy sample was taken from the regenerated cartilage, and fairly thick regenerated cartilage was recognizable at the biopsy site.

**Figure 2:**
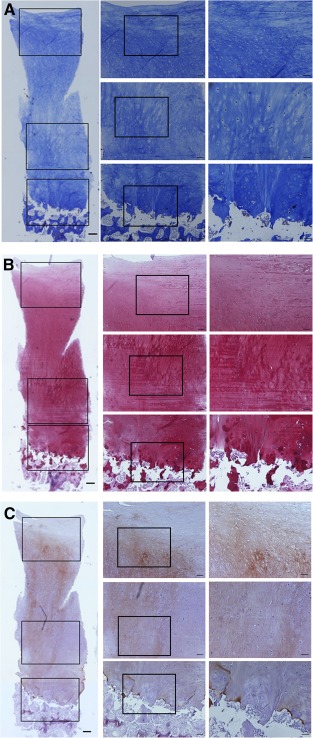
Histological findings of the regenerated cartilage. The histologic analysis of the biopsy sample showed that the regenerated cartilage had a staining pattern similar to that of normal articular hyaline cartilage. A columnar alignment of chondrocytes with lacunae formation was observed at the regenerated cartilage. **(A):** Masson's trichrome staining showed abundant collagen staining. **(B):** Safranin O staining showed abundant glycosaminoglycan staining. **(C):** Immunohistochemical staining showed strong staining of type II collagen, which is typical in articular hyaline cartilage. The superficial region near the articular surface is less strongly stained with Masson's trichrome, Safranin O, and type II collagen antibody, and the deepest portion of the repair site was transformed to a subchondral bone, which is similar to the histological architecture of native articular cartilage. Scale bars = 200 µm (left), 100 µm (middle), and 50 µm (right).

Maturing repair tissue was observed at the 12‐week arthroscopic examination (supplemental online Table 1). In the extended follow‐up, two participants consented to the arthroscopic examination with biopsy at 1 year. The arthroscopic examination at 1 year revealed good resurfacing with thick and glossy white hyaline‐like cartilage at the lesion site. The regenerated cartilage had a smooth surface with firm consistency and also showed good integration with the surrounding native cartilage (Fig. 1). No bone formation or overgrowth was observed. The biopsy specimens were stained strongly with Masson's trichrome, Safranin O, and the immunohistochemical stain for type II collagen, which represents a similar histology to that of native hyaline cartilage (Fig. 2).

**Figure 3:**
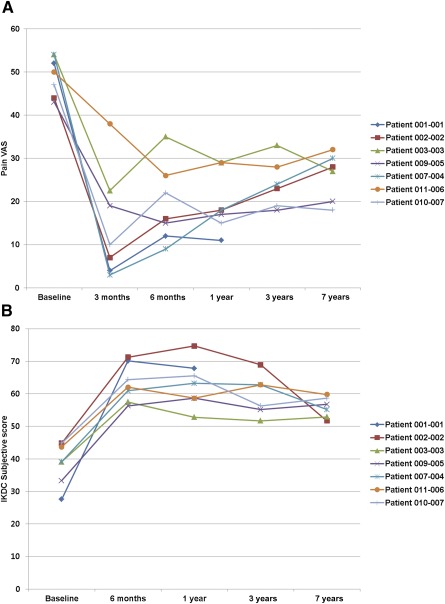
Changes in VAS score for pain and IKDC subjective knee scores over the 7‐year follow‐up period. **(A):** The VAS score for pain was significantly improved in all participants at 3 months post‐transplantation (a result that may have been significantly affected by nonweight‐bearing ambulation for that period) and increased nonsignificantly at 6 months (*p* = .204). The improvement in pain at 6 months was maintained for 7 years. **(B):** The IKDC subjective knee score was improved significantly in all participants at 6 months. This improvement did not deteriorate significantly over 7 years. Abbreviations: IKDC, International Knee Documentation Committee; VAS, visual analog scale.

The 100‐mm VAS score for pain improved from 49.1 at pretransplantation to 19.3 at 24 weeks post‐transplantation (*p* = .018). The IKDC subjective score also improved from 39.1 at pretransplantation to 63.2 at 24 weeks post‐transplantation (*p* = .018) in all seven participants. These improved scores were maintained without significant deterioration up to 7 years (Fig. 3).

**Figure 4:**
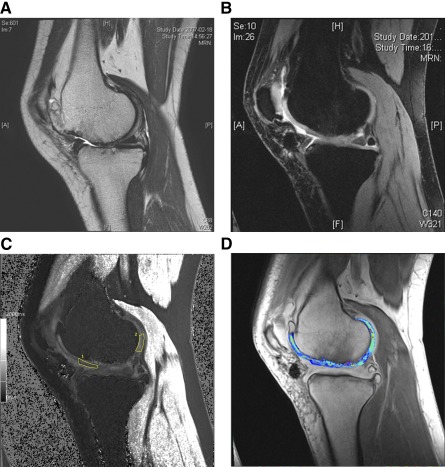
Magnetic resonance imaging (MRI) evaluation of cartilage regeneration 3 years after human umbilical cord blood‐derived mesenchymal stem cell‐hyaluronic acid hydrogel composite transplantation. **(A):** A cartilage defect of International Cartilage Repair Society grade 4 was detected at the medial femoral condyle by a preoperative MRI. **(B):** Cartilage regeneration at the defect site was observed at the medial femoral condyle at 3 years post‐transplantation. **(C):** The change in relaxation rate (ΔR1) in regenerated cartilage and ΔR1 in native cartilage were obtained at the marked areas to calculate the relative ΔR1. **(D):** A high glycosaminoglycan (GAG) content was observed in the regenerated cartilage by delayed gadolinium‐enhanced MRI of the cartilage. Higher T1 values (blue) are associated with increased relative GAG content, whereas lower T1 values (red) are associated with decreased GAG content.

Five participants consented to MRI evaluation at 3 years. The mean relative ΔR1 index was 1.44 (supplemental online Table 2), which indicated high GAG content of the regenerated cartilage (Fig. 4).

## Discussion

The results of this phase I/II clinical trial show that a novel stem cell‐based medicinal product (Cartistem), composed of allogeneic hUCB‐MSCs and HA hydrogel, had an acceptable efficacy and safety profile during the core trial phase and over extended follow‐up. No significant AEs or undesired effects such as overgrowth, osseous metaplasia, or tumorigenesis were observed during this study. One TEAE was reported (elevation of the antithyroglobulin antibody level) but normalized spontaneously. Arthroscopic examinations at 1 year showed well‐healed cartilage defect sites. The histological assessment at 1 year showed highly hyaline‐like cartilage. A dGEMRIC at 3 years showed persistent regenerated cartilage with high GAG content. The improved pain and function of participants did not show significant deterioration over 7 years of follow‐up.

To our knowledge, this is the first report of a clinical trial investigating an allogeneic stem cell‐based medicinal product (allogeneic hUCB‐MSC‐based therapeutics) for the regeneration of articular cartilage defects in osteoarthritic patients. As mentioned, there have been some studies that explored the application of stem cell therapy on osteoarthritis. However, upon closer inspection, these studies, for the most part, describe results of medical procedures using a heterogeneous cell population obtained from an autologous tissue containing an uncertain number of stem cells 7, 8, 9. The cell population obtained by these medical procedures contains variable numbers of autologous stem cells, which is less than 0.1% in the case of the bone marrow aspirate concentration procedure 20. We question whether the term “stem cell therapy” is acceptable for this kind of medical procedure containing only a small number of stem cells 8.

Recently, there have been a few reports of actual stem cell therapy using autologous stem cells (a homogeneous stem cell population that was isolated from autologous tissue and expanded in culture) for the regenerative treatment of osteoarthritic cartilage defects 10, 11, 12. However, clinical use of allogeneic stem cells for cartilage regeneration has not been reported and, therefore, information on efficacy and safety is lacking. Although autologous BM‐MSCs have been extensively studied, the collection of autologous BM‐MSCs is invasive. More importantly, the number of MSCs obtained from harvested BM is very limited, especially in older patients 20. The hUCB‐MSCs have a higher proliferation rate, karyotype stability after prolonged expansion, and greater chondrogenic potential compared with BM‐MSCs 21. Moreover, hUCB‐MSCs have several additional advantages, including ease of availability from cord blood banks, noninvasive collection procedures, and hypoimmunogenic properties 22. There have been some preclinical studies that showed efficacy of hUCB‐MSCs for cartilage regeneration 15, 23, 24. Thus, we believe that hUCB‐MSCs are strong candidates for development of a novel stem cell‐based medicinal product for cartilage regeneration in osteoarthritic patients.

Our study reveals that treatment with an allogeneic hUCB‐MSC product (Cartistem) is safe. Our results show no significant AEs or undesired effects over 7 years of follow‐up. None of the participants had substantial permanent morbidity, nor did any subjects discontinue the study prematurely after transplantation. Only one participant was lost in the long‐term extension phase, because she died of senile infirmity at 6 years post‐transplantation at the age of 76. We also found no evidence of abnormal findings suggesting rejection or infection. We believe that the characteristics of the hUCB‐MSCs such as hypoimmunogenicity and immunomodulatory activity 25, 26 contributed to the lack of rejection, despite their allogeneic use.

Treatment with the new hUCB‐MSC–HA composite was associated with no detectable immunological issues and resulted in successful regeneration of articular cartilage, even in immunocompetent animals 15, 23, 24. The transplanted cells disappeared gradually from 4 to 8 weeks in rabbit and mini‐pig models 24. Other in vivo studies that investigated the fate of transplanted MSCs in osteoarthritis reported that labeled transplanted MSCs gradually disappeared in the regenerated tissue over time 27. We believe that the gradual disappearance of transplanted hUCB‐MSCs contributes to the long‐term safety of allogeneic transplantation of hUCB‐MSCs that we observed in our study. Currently, the mode of action of hUCB‐MSCs is understood to promote differentiation of endogenous chondroprogenitor cells by paracrine action 28. These findings support the use of allogeneic hUCB‐MSCs as a safe therapeutic option in the clinical setting that warrants further investigation to confirm the safety and efficacy in a larger patient population.

The results of this study also suggest that the novel allogeneic stem cell‐based medicinal product results in good‐quality cartilage regeneration in osteoarthritis. The histological evaluation at 1 year revealed that the repair tissue was similar to native hyaline cartilage. At the 3‐year MRI assessment for the quality of repaired tissue, the average relative ΔR1 was 1.44 among the five participants studied, which suggests that the repaired tissue is comparable to healthy native cartilage. Previous reports indicate that relative ΔR1 was 1.32 at 1 year post‐ACI for focal chondral defects in nine patients (average age, 21.2 years) 19, and 1.13 and 1.55 at 1 year and 2 years, respectively, for postosteochondral allograft transplantation in nine patients (average age, 43.2 years) 29. Although the hUCB‐MSCs‐HA composite was transplanted at sites of osteoarthritic cartilage defects in elderly patients in this study, the relative ΔR1 of this study was comparable with that after ACI or osteochondral allograft transplantation in younger patients with focal chondral defects 19, 29. These results suggest that hUCB‐MSC–HA hydrogel composite transplantation may have a more favorable outcome than currently available cartilage repair methods.

Another notable finding of this study is that the improvements in pain and function at 24 weeks post‐transplantation did not deteriorate significantly for 7 years. In addition, the participants enrolled in this study were patients with K‐L grade 3 osteoarthritis at baseline, but did not undergo any additional knee surgery or knee joint‐replacement surgery during the 7‐year follow‐up period post‐transplantation. These long‐term results are significant given that microfracture has been reported to provide short‐term improvement in knee pain and function for osteoarthritic cartilage lesions, but cartilage once again deteriorates after 1–2 years 30. This decline is attributed to the difference in durability of the fibrocartilage that results from microfracture compared with native hyaline cartilage 3. Therefore, the findings of our study are encouraging because current articular cartilage repair procedures are known to be unsatisfactory among elderly patients with large defects 5, 31, 32. In the present study, the repair site was restored with hyaline‐like cartilage tissue, which seemed to contribute to the observed persistence of the regenerated cartilage with durable improvement in pain and function. These findings suggest that the hUCB‐MSC–HA composite can be a stem cell‐based therapeutic product for the treatment of osteoarthritic cartilage defects.

Limitations of this study should be considered. First, a small number of patients participated in this clinical trial. Small sample size is an inherent limitation of this type of phase I/II first‐in‐human clinical trial because the risk‐benefit ratio in humans is unknown. This study included surgical procedures and was the first in‐human clinical trial of the allogeneic UCB‐MSC product. Thus, the regulatory authority only allowed a small number of patients to participate with a dose‐escalation protocol. Second, there is no control group in this clinical trial. Because there was no optimal method for cartilage regeneration in osteoarthritis that could have been used as a control procedure and there were also concerns about making large‐bore drill holes (5 mm) at the subchondral bone without any additions, this clinical trial could not have been conducted with a control group. The microfracture technique using small‐bore drill holes (usually 1–2 mm) has resulted in deterioration within a few years 2, 3. Moreover, in our preclinical studies with hUCB‐MSCs, we found that hUCB‐MSC transplantation showed favorable cartilage regeneration compared with an untreated defect‐only control group 23, 24. Although this study has no control group (defect only), the safety and efficacy of hUCB‐MSCs for cartilage regeneration were verified in the long‐term extended follow‐up period. Third, this study could not evaluate cartilage repair in highly deformed knees. The participants in this study had K‐L grade 3 osteoarthritis; therefore, the lower extremity alignment was not severely deformed. Fourth, only two participants consented to the arthroscopic examination for biopsy at 1‐year post‐transplantation. The dGEMRIC, however, was performed in five participants, and demonstrated encouraging results in terms of quality of the repair tissue. Finally, the mode of action of the hUCB‐MSCs for the regeneration of articular cartilage is only partially understood 28.

## Conclusion

Application of the allogeneic hUCB‐MSCs‐based novel medicinal product appears to be safe and effective for the regeneration of durable hyaline‐like cartilage in osteoarthritic knees. The results of this study warrant further investigation of this novel therapeutic product in a larger number of patients.

## Author Contributions

Y.‐B.P. and C.‐W.H.: conception and design, collection and/or assembly of data, data analysis and interpretation, manuscript writing, final approval of the manuscript; C.‐H.L. and Y.C.Y.: collection and/or assembly of data, data analysis and interpretation; Y.‐G.P: collection and/or assembly of data, data analysis and interpretation, manuscript writing.

## Disclosure of Potential Conflicts of Interest

C.‐W.H. received grant support from the Korea Health Industry Development Institute, funded by the Ministry of Health and Welfare, Republic of Korea, and from Medipost Co. during the conduct of this study. The other authors indicated no potential conflicts of interest.

## Supporting information

Supporting Information
