# Supplementary material for: Cartilage Regeneration in Osteoarthritic Patients by a Composite of Allogeneic Umbilical Cord Blood‐Derived Mesenchymal Stem Cells and Hyaluronate Hydrogel: Results from a Clinical Trial for Safety and Proof‐of‐Concept with 7 Years of Extended Follow‐Up
Source: Stem Cells Transl Med. 2016 Sep 9;6(2):613–21. doi: 10.5966/sctm.2016-0157 (PMC5442809; doi:10.5966/sctm.2016-0157)
Supplement: Supplementary file 1 — Supporting Information [file SCT3-6-613-s001.pdf]

Supplemental Information – Ha et al.

Figure S1. Transplantation of the hUCB-MSCs-HA hydrogel composite

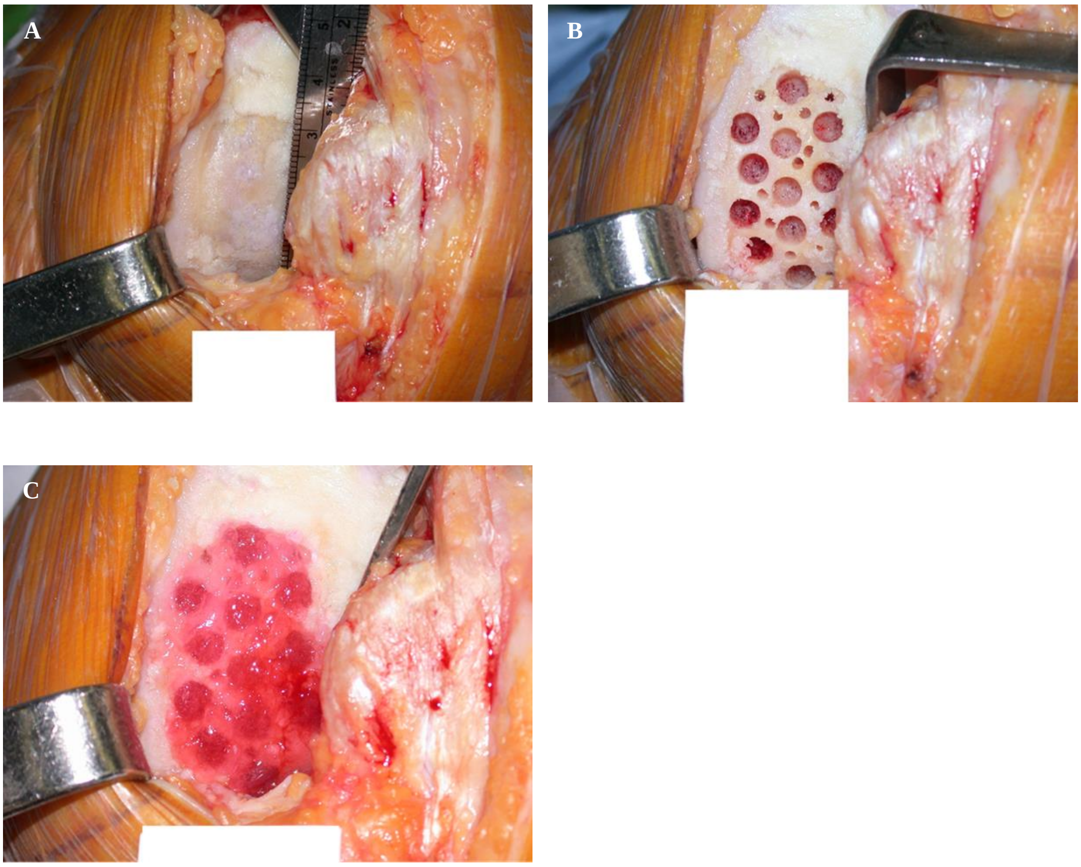

(A) The cartilage defect site was identified as a completely worn out cartilage lesion with partial erosion of the subchondral bone (ICRS grade 4). (B) Multiple drill holes (5 mm in diameter and 5 mm in depth) were made at the lesion site. In addition, multiple drilling with a 1.4 mm K-wire was performed between the 5 mm diameter drill holes. (C) The hUCB-MSCs-HA hydrogel composite was implanted into the 5 mm drill holes and on the surface of the lesion.

Supporting Information Figure 2. CONSORT flow diagram

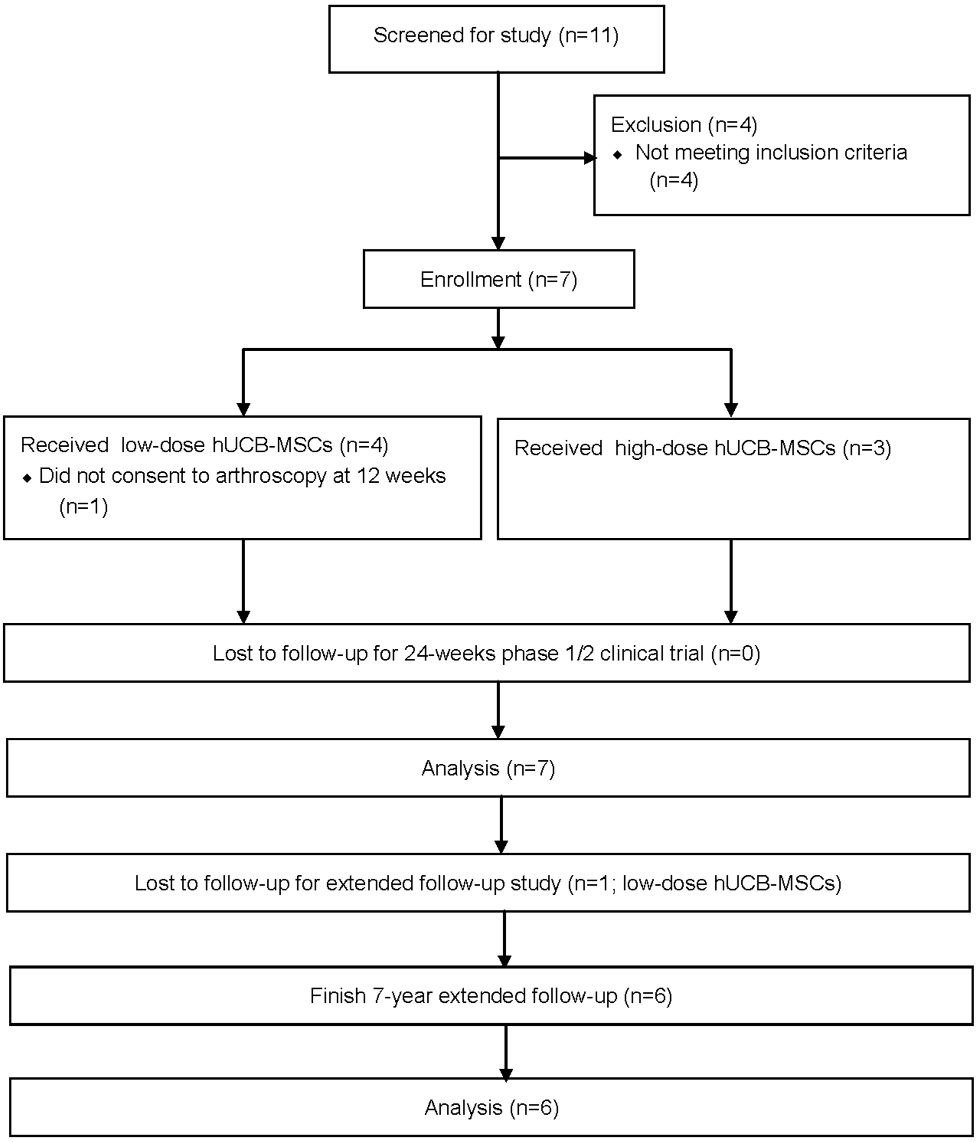

Schematic of patient screening, enrollment, and completion of the trial.

Supporting Information Table 1. ICRS cartilage repair assessment by arthroscopic examinations

Group (A: low dose, B: high does)	ID	Age (years)	Gender	Defect size (cm^2)	ICRS grade of cartilage lesion (pre-transplantion)	ICRS cartilage repair assessment (12 weeks post-transplantion)	ICRS cartilage repair assessment (1 year post-transplantion)
A	001-001	68	F	5.0	4	Did not consent	Did not consent
A	002-002	66	M	4.8	4	3	Did not consent
A	003-003	58	F	5.0	4	3	Did not consent
A	009-005	29	F	4.6	4	2	Did not consent
B	007-004	62	F	6.6	4	3	2
B	011-006	51	M	8.1	4	4	3
B	010-007	77	F	7.1	4	4	Did not consent

ICRS, International Cartilage Repair Society

Supporting Information Table 2. MRI evaluation of pre- and post-contrast R1 and ΔR1 in native and reparative cartilages at 3 years post-transplantation

Group (A: low dose, B: high does)	ID	Regenerative cartilage			Native cartilage			Relative R1 index
		R1pre (1/s)	R1post (1/s)	ΔR1	R1pre (1/s)	R1post (1/s)	ΔR1	
A	001-001	-	-	-	-	-	-	Not consented
A	002-002	0.96	1.85	0.89	1.29	1.86	0.57	1.56
A	003-003	-	-	-	-	-		Not consented
A	009-005	1.15	2.49	1.34	0.94	1.72	0.78	1.72
B	007-004	0.91	3.08	2.17	0.99	2.37	1.38	1.57
B	011-006	1.02	3.19	2.17	0.96	2.69	1.73	1.25
B	010-007	0.84	2.32	1.48	1.06	2.43	1.37	1.08
Average*		0.98 (0.12)	2.59 (0.55)	1.61 (0.56)	1.05 (0·14)	2.21 (0.41)	1.17 (0.48)	1.44 (0.26)

*Data are mean (standard deviation).
